# Getting Road Expansion on the Right Track: A Framework for Smart Infrastructure Planning in the Mekong

**DOI:** 10.1371/journal.pbio.2000266

**Published:** 2016-12-15

**Authors:** Andrew Balmford, Huafang Chen, Ben Phalan, Mingcheng Wang, Christine O’Connell, Cath Tayleur, Jianchu Xu

**Affiliations:** 1 Conservation Science Group, Department of Zoology, Cambridge, United Kingdom; 2 Key Laboratory of Plant Diversity and Biogeography of East Asia, Kunming Institute of Botany, Chinese Academy of Sciences, Kunming, China; 3 World Agroforestry Center, East and Central Asia, Kunming, China; 4 Department of Forest Ecosystems and Society, Oregon State University, Corvallis, Oregon, United States of America; 5 Department of Environmental Science, Policy, and Management, University of California, Berkeley, Berkeley, California, United States of America; 6 RSPB Centre for Conservation Science, The Royal Society for the Protection of Birds, Sandy, United Kingdom; University College London, United Kingdom of Great Britain and Northern Ireland

## Abstract

The current unprecedented expansion of infrastructure promises to enhance human wellbeing but risks causing substantial harm to natural ecosystems and the benefits they provide for people. A framework for systematically and proactively identifying the likely benefits and costs of such developments is badly needed. Here, we develop and test at the subregional scale a recently proposed global scheme for comparing the potential gains from new roads for food production with their likely impact on biodiversity and ecosystem services. Working in the Greater Mekong—an exceptionally biodiverse subregion undergoing rapid development—we combined maps of isolation from urban centres, yield gaps, and the current area under 17 crops to estimate where and how far road development could in principle help to increase food production without the need for cropland expansion. We overlaid this information with maps summarising the importance of remaining habitats to terrestrial vertebrates and (as examples of major ecosystem services) to global and local climate regulation. This intersection revealed several largely converted yet relatively low-yielding areas (such as central, eastern, and northeastern Thailand and the Ayeyarwady Delta), where narrowing yield gaps by improving transport links has the potential to substantially increase food production at relatively limited environmental cost. Concentrating new roads and road improvements here while taking strong measures to prevent their spread into areas which are still extensively forested (such as northern Laos, western Yunnan, and southwestern Cambodia) could thus enhance rural livelihoods and regional food production while helping safeguard vital ecosystem services and globally significant biological diversity.

## Author Summary

Proposals for new infrastructure in developing countries are typically muted on its environmental impacts, while environmentalists typically say little about its potential benefits for people. This study explores a more conciliatory approach by trying to identify where beneficial infrastructure might be expanded at least environmental cost. Focusing on the Greater Mekong Subregion of Southeast Asia, we intersected agricultural, social, and environmental layers to map variation in the potential for new roads to enhance food production and in their likely impacts on biodiversity and ecosystem services. In several areas (such as central Thailand and the Ayeyarwady Delta) well-planned road expansion has the potential—if linked to strong habitat protection elsewhere—to help boost agricultural production at limited environmental cost. In others (including much of Laos, Central Vietnam, and eastern Cambodia), new roads would risk marked environmental harm, often for little agricultural gain. By mapping specific proposals onto our data layers, we were also able to identify planned roads that might deliver low-impact improvements in food production and others that risk disproportionate environmental costs. We hope these analyses can help guide future road planning in this exceptionally diverse, rapidly developing area.

## Introduction

The world is undergoing an unprecedented expansion of human infrastructure. For example, more than 450 new hydropower dams are planned for the Amazon, Congo, and Mekong basins alone [1]. Likewise, a recent report from the International Energy Agency states that by 2050 the world will build an estimated 335,000 km of new railway tracks and 25 million km of new road lanes—the great majority in developing countries [2,3]—while the newly established Asian Infrastructure Investment Bank alone has already built up US$100 billion in capital for investments in the Asia-Pacific region, and the New Development Bank has starting capital of US$50 billion [4]. Road building is a top priority for China’s “One Belt One Road” development initiative [5].

New infrastructure can bring substantial benefits to people [6–10] but if poorly planned can impose very significant environmental costs. New roads in wilderness areas, for example, often (and sometimes deliberately) stimulate habitat conversion for farming and mining and facilitate overhunting, unsustainable logging, and wildfires [11–14]. Existing approaches for assessing environmental impacts typically overlook these cascading effects and are hampered by poor data and a tendency to place the burden of proof on environmental organisations rather than developers [15–17]. There is instead a need to develop a robust, spatially explicit, and proactive framework for estimating probable environmental costs of planned infrastructure and weighing these up against likely benefits [18].

Infrastructure development—especially road construction—is often aimed at enhancing food production and reducing poverty. New and upgraded roads can reduce the transport costs of agricultural inputs and of getting products to market, lower postharvesting food waste, and increase access to technological improvements [6,19–22]. To the extent that farming becomes more profitable (or demand is stimulated by falling food prices), this can in turn accelerate conversion of natural habitats to agriculture. However, if new roads are deployed strategically—deliberately targeting already cleared areas with poor transport connectivity and attracting agricultural growth that might otherwise spread elsewhere, within a context of well-enforced land-use zoning—they could in principle play an important role not just in increasing food production but in safeguarding unconverted areas of high environmental value [19,23]. Viewed this way, judicious road planning is a potentially promising mechanism for delivering land sparing: boosting farm yield (production per unit area) while at the same time protecting or restoring natural habitat on other land that is then not needed for meeting agricultural demand [18,24–26].

This recognition of the potential gains from promoting new roads in places where they can most enhance farming while avoiding highly sensitive areas has been formalised in a new “global road-mapping strategy” [15]. In principle, the approach is applicable to limiting the damage caused by infrastructure built for any need (such as accessing natural resources or improving human health), but the risks and opportunities are especially clear in the context of roads and food production. Working at a global scale, this framework therefore proposes combining information from 21 different layers to derive aggregate surfaces describing spatial variation in the potential food production gains and environmental costs of new or improved roads. Based on these surfaces, areas can then be identified where road development might enable marked food production benefits at moderate environmental cost, where new roads would probably do little to increase production but risk considerable ecological damage, where both costs and benefits would probably be limited, and where both might be high and hence agricultural and environmental priorities might be in conflict. However, while this exercise is a useful illustration of how in principle prospective benefits and environmental costs might be systematically compared, roads are not planned at a global scale. To be practical, the framework needs to be applied and tested at finer scales—nearer to those at which road planning does occur—and linked to real-world road proposals.

To address this gap, our paper adopts, refines, and tests the approach of the global road-planning strategy to examine its potential to contribute to finer-scale decision making on road investment, with three overarching aims:

1. To examine the generality and practicality of the approach by applying it to a highly biodiverse but rapidly developing subregion—the Greater Mekong (Materials and Methods)—rather than at a global [15] or continental [27] scale), and by refining the methods used to generate aggregate surfaces of potential food production benefits and environmental costs.
2. To explore the sensitivity of the results to the data layers used to create these surfaces, to how they are combined, and to the type of benefits from road development which they consider.
3. To use the framework to characterize specific roads which have been proposed as development priorities in the Greater Mekong Subregion (GMS) and to identify a subset that seem well situated to address poverty and food production concerns at potentially limited environmental cost and others that appear less beneficial.

## Results

### Potential Food Production Benefit

**Fig 1:**
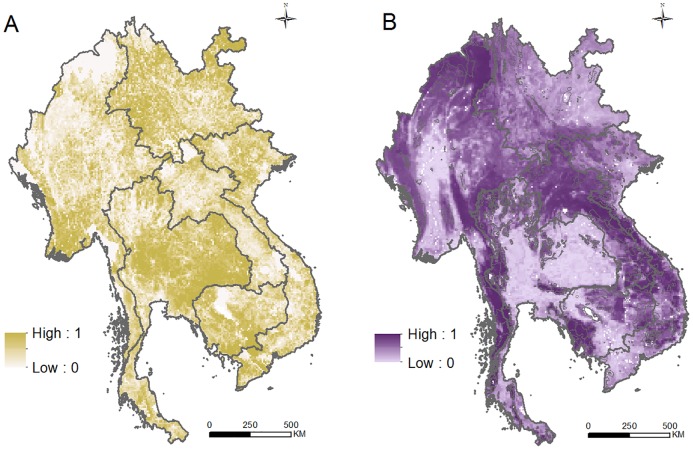
Potential benefits and costs of new roads. Aggregate surfaces of potential food production benefit (A) and potential environmental cost (B) from road development, plotted in ten equal-area intervals. Potential benefit was calculated by multiplying the additional food energy that could be produced by closing yield gaps by a measure of isolation for each grid cell. Potential cost was calculated as the mean of each cell’s importance for terrestrial vertebrates, for carbon storage, and for climate regulation. In (B), protected areas are outlined in black. Underlying data can be found in S1 Data.

Our analysis suggested substantial potential for increasing food production in the GMS through closing existing yield gaps, without any change in the area under production. Across the 17 crops we considered, increasing yields to those attained in climatically similar areas [28] could raise annual production by ~120 million tonnes (or in food energy terms, 1.77 billion gigajoules, an increase of 57% over current levels). Production gaps (Materials and Methods) appear greatest around Myanmar’s Ayeyarwady Delta, a large swathe of central, eastern, and northeastern Thailand, in northeastern Yunnan, and in southwestern Vietnam (S1A Fig). Taking isolation into account to build our aggregate surface of the potential food production benefit of more roads had little effect on this overall pattern, except close to major cities (Fig 1A). The aggregate surface suggested road enhancement has the least potential to increase food production in northern Myanmar and northwestern Yunnan, parts of northern and southern Thailand, and in Laos, central Vietnam, and eastern Cambodia.

### Potential Environmental Cost

Almost the entire GMS falls within the Indo-Burma Hotspot [29], and as such, it is globally significant for conservation. However, even within the subregion, environmental values vary. The importance of remaining natural vegetation in each of our three environmental layers (Materials and Methods) (S3–S5 Figs) meant that they picked out a common set of relatively unconverted areas where new roads could cause particular environmental harm (Fig 1B): northern and western Myanmar, the length of the Thai and Myanmar border, much of Laos and central Vietnam, and eastern and southwestern Cambodia. In general, these areas correspond reasonably well with established conservation priorities, including most but not all of the GMS’ protected areas (Fig 1B) [30], Important Bird and Biodiversity Areas [31], Endemic Bird Areas [32], Centres of Plant Diversity [33] and Frontier Forests [34], and all but one of the biodiversity conservation landscapes of the Asian Development Bank (ADB) [35]. Comparison with other conservation templates is less informative, as they prioritize almost all the region (Biodiversity Hotspots [36]; Global 200 Ecoregions [37]) or none of it (High-Biodiversity Wilderness Areas [38]).

### Intersecting Potential Benefit and Cost

**Fig 2:**
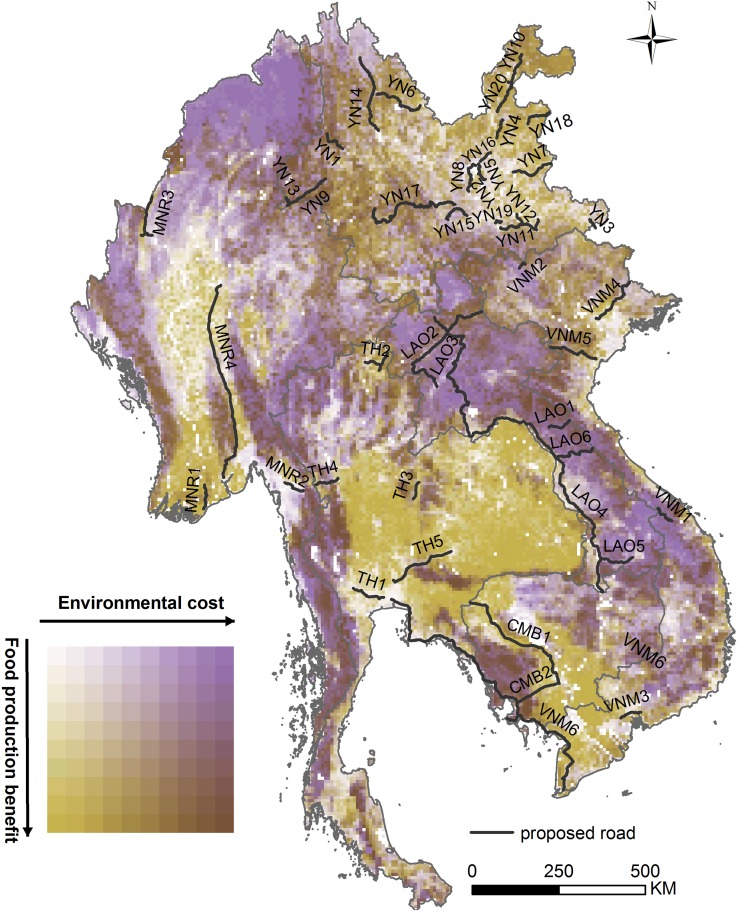
Benefits and costs compared. Intersection of surfaces describing the potential food production benefit and environmental cost of road development, derived by overlaying aggregate surfaces mapped in Fig 1. Proposals for specific roads are superimposed in black (see S2 Table). The white area in central Cambodia is Lake Tonlé Sap. Underlying data can be found in S1 Data.

Overlaying our aggregate surfaces suggested that the balance of likely environmental costs and food production benefits from road growth varies widely across the GMS (Fig 2). Areas with few rural roads presently (Materials and Methods) and where investment in transport infrastructure might boost food production considerably at potentially low relative environmental cost (plotted in olive green; lower left in Fig 2 inset) include the Ayeyarwady Delta; much of central, eastern, and northeastern Thailand; parts of northeast Yunnan; and a band from central Cambodia to southwestern Vietnam. In contrast, areas where food production gains from new roads would probably be modest yet carry high environmental risk (in purple; upper right in Fig 2 inset) include most of Myanmar outside the Ayeyarwady Delta as well as most of Laos, central Vietnam, and eastern Cambodia. Food production gains might also be limited but environmental risks lower (indicated by paler shades; upper left in Fig 2 inset) in areas such as the Ayeyarwady Plains (although the few habitat fragments still remaining there have high conservation value [39]). Potential conflicts appear most pronounced in environmentally sensitive areas with marked scope for increased food production (shown in the darkest shades; lower right in Fig 2 inset), such as parts of western Yunnan, southern Myanmar, extreme western and northern Thailand, the border area between northern Laos and Vietnam, and southwestern Cambodia.

### Sensitivity Analyses

**Fig 3:**
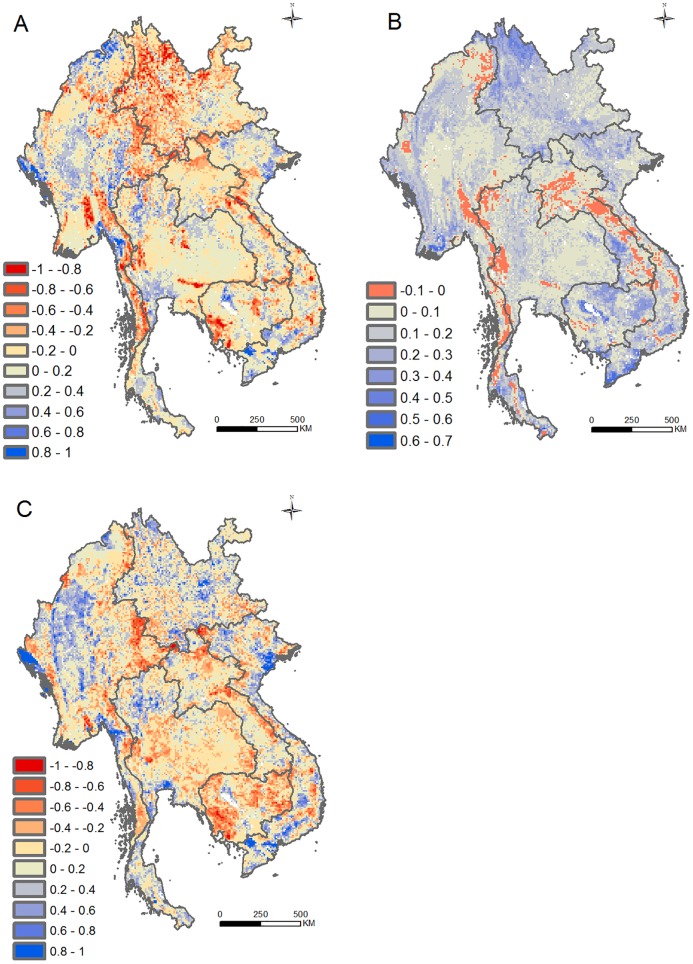
Sensitivity tests. Results of three tests of the sensitivity of our aggregate benefit and cost surfaces to the data and rules used to assemble them: the difference in benefit scores if food production benefit was estimated using different data on yield gaps ([40] cf. [28]) (A), the difference in cost scores if they were based on the maximum of the three component values in any cell rather than their mean (B); and the difference in benefit scores if they were based on the product of human population density and isolation rather than potential food production benefit (C). In each case, the difference is expressed as the new minus the original aggregate score. Red colours thus correspond to lower benefit or cost scores (and blue to higher scores) than those mapped in Fig 2. Underlying data can be found in S1 Data.

The data layers we used inevitably have errors, were compiled for different purposes, and could defensibly be integrated in other ways than we have considered here. However, three sorts of sensitivity tests (Materials and Methods) indicated that our overall assessment of the relative benefits and costs of road development in the GMS was moderately robust to using different data layers and rules for combining them. When we used a different yield gap map (Fig 3A), took as our aggregate cost surface the maximum (rather than mean) of the three underlying environmental layers (Fig 3B), and constructed a new potential benefit surface based on the distribution of people rather than yield gaps (Fig 3C), the respective benefit and cost values changed by >ǀ0.2ǀ for a sizeable number (though still less than half) of all grid cells (for 43%, 20%, and 48% of cells, respectively). The characterization of different areas in our dual-colour overlay changed rather little overall, although there were a few noteworthy shifts. For example, employing a different yield gap measure lowered our estimate of food production benefit (red in Fig 3A) and hence lessened potential conflict (dark in Fig 2) in parts of central Myanmar, central Thailand, western Yunnan, eastern Laos, and southwestern Cambodia. A similar reduction in conflict emerged when population (rather than agricultural) gains from new roads were considered, with road benefits increasing around major cities (blue in Fig 3C) and declining (red in Fig 3C) in potential conflict areas (dark in Fig 2) in eastern Myanmar, southwestern Cambodia, and northwestern Vietnam.

### Specific Road Proposals

Despite the relatively coarse resolution of our analysis, overlaying the locations of 43 road development schemes onto our intersection of the potential benefits and costs of new roads (Fig 2, S8A Fig and S2 Table) can shed light on the merits of specific road proposals. Thus, while the potential impacts of individual schemes often vary along their length (S8B and S8C Fig), in broad terms some roads (e.g., YN18 and YN20 in Yunnan; MNR1 in Myanmar; CMB1 in Cambodia; VNM3 in Vietnam, all in the lower left part of S8A Fig) could perhaps enhance food production at limited environmental cost (although some of these are in areas already relatively well served by roads [S6 Fig]). On the other hand, several proposals (e.g., roads YN1, YN9, YN14, and YN17 in Yunnan; parts of MNR3 and MNR4 in Myanmar; TH4 in Thailand; parts of CMB2 in Cambodia; LAO1-6 in Laos; VNM1, VNM2, and VNM5 in Vietnam) have the potential to be especially harmful to environmental values at the regionwide scale; most of these (e.g., YN1, YN14, MNR4, TH4, CMB2, LAO1, LAO4-6, VNM1, VNM2, and VNM5) would even cut through or run next to established protected areas. It is striking that a number of these high-risk developments (e.g., roads LAO1, LAO3, LAO5, LASO6, VNM1, and VNM2 in the upper right of S8A Fig) are in areas where agricultural gains would apparently be limited, while the likely benefits of some others (e.g., roads YN1, YN9, YN14, MNR4, CMB2, and LAO2) are lower if alternative benefit layers are considered (Fig 3A and 3C). Many of these potentially most-damaging developments (e.g., roads YN1,YN14, YN17, TH4, CMB2, LAO1, LAO4-6, and VNM2) also run through or close to the ADB’s biodiversity conservation landscapes—recognised by the Bank as being “globally high-value landscapes” that “need to be conserved to safeguard local livelihoods and investments in energy…water and sectors that enhance food security” [35].

## Discussion

Our analyses indicate that even within a global biodiversity hotspot (characterised by extensive habitat conversion and exceptional biological value [41]) there is scope, in principle, for well-planned road enhancement to boost agricultural production at relatively limited environmental cost. This result was not apparent in a previous global-scale analysis (Fig 3 in [15]), in part because in the present study, environmental costs were scaled against broadly high subregional values (cf. lower global ones in [15]); this is appropriate given the need to identify lower-cost options for lifting food production in an area with rapid population growth and marked poverty. But two other reasons for our result are more interesting. First, there are significant gaps in crop yields across much of the GMS, where production could be increased without expanding cropland area. Second, the extent of habitat conversion, most of it to cropland, varies quite considerably. Highly converted areas (of consequently low relative value for biodiversity and carbon-based ecosystem services) often have marked yield gaps, whereas areas of largely intact habitat have little farmland (and hence limited potential for production to be increased without cropland expansion). This creates a broadly negative spatial association between our food production benefit and environmental cost surfaces (borne out by the relative scarcity of light and dark cells in Fig 2) and means that strategic road development has the potential to contribute to land sparing in the GMS [26].

Nevertheless, considerable care is required to realise this potential. In many areas (shown in purple in Fig 2), road expansion is unlikely to boost agriculture unless forests of very high environmental value are cleared for new cropland. In other areas (e.g., in western Yunnan, western and northern Thailand, and southwestern Cambodia, shown dark in Fig 2), roads might help raise yields but would also risk pronounced damage to biodiversity and climate regulation. Road proposals here should be subject to particular scrutiny, with mitigation likely to be both difficult and costly. Instead, we suggest that road investments aimed at improving agricultural livelihoods should focus on areas of high likely benefit and low potential cost (olive green in Fig 2). These occur in every country in the region apart from Laos. Even within these areas, though, care should be taken to avoid road development in intact habitat fragments which may be highly valuable yet too small to be identified in analyses at this scale.

These general rules of thumb are subject to four sets of caveats:

1. The agricultural layers we used are imperfect and far from comprehensive. Our yield gap information deals with only 67% of cropland area, excludes regionally important crops such as rubber and oil palm and is based on currently attainable yields—so it is likely to prove conservative as new varieties and practices raise yield ceilings. More broadly, while there is good empirical evidence that road enhancement can boost farm production [5,15–18] and help address poverty, in assessing specific proposals it would be important to have direct, local information on how far yields are indeed limited by transport costs, premarket food waste, and existing road quality rather than factors (such as management practices or seed quality) that are less readily resolved by enhanced access.
2. Likewise, our environmental layers convey no direct information on essential ecosystem services for local communities such as flood regulation or provision of harvestable fish and include no data on invertebrates, plants, or freshwater biodiversity. Lack of information means we have also not looked at other potentially harmful impacts of roads—such as facilitating the expansion of hunting, increasing soil erosion, and lowering connectivity between remaining natural areas. These risks would be hard to map across the entire GMS but should be considered for specific road proposals on a case-by-case basis.
3. While the scale of our analysis is relevant to subregion-wide planning efforts by the Asian Development Bank and the newly established Asian Infrastructure Investment Bank and uncovers patterns not apparent at the global level, finer-scale assessments involving thorough environmental and social impact analyses are of course still needed [16]. These are likely to be important in identifying more (and less) sensitive portions of individual road schemes, small but environmentally important patches in largely converted areas (such as central and northeastern Thailand), and locations where conversion has already lowered the likely costs of yield improvement within areas of otherwise high environmental value (such as Laos). If fine-scale planning is conducted in participation with local communities, it could also help safeguard the subregion’s rich cultural diversity.
4. Crucially, directing road investments into agriculturally rewarding areas of relatively low environmental value cannot by itself deliver land sparing. To be effective in limiting the costs of meeting rising food demand while avoiding the cascading negative consequences of road expansion, strategic infrastructure deployment must be coupled with enhanced protection of high-value landscapes elsewhere through land-use zoning, protected areas, ecological restoration, or other interventions [26]. Simply leaving such places with limited infrastructure but without explicit regulatory protection is unlikely to be sufficient to safeguard them into the future.

While our results should therefore be interpreted with caution, we suggest they illustrate how the framework of the global road-building strategy can be adapted and applied at scales relevant to decision makers (for an intermediate, continent-level analysis, see [27]). Our findings here are based on layers available for the entire world, they are moderately robust to quite substantial differences in which data layers and combinatorial rules are used, and they shed new light on the scope for moderate-impact, road-mediated increases in agricultural production in the GMS. We believe that the general approach of trying to make spatially explicit the trade-offs between potential benefits of new infrastructure and their likely environmental costs may be helpful in other contexts too. It could be applied to roads proposed for other reasons, such as improving transport of minerals to ports, enhancing tourism, or enabling access to militarily sensitive areas. In an agricultural context, if information on the sensitivity of freshwater ecosystems could be acquired, our scheme could be extended to explore where additional irrigation (whose likely impacts on yield have already been mapped—Fig 5B in [28]) might be developed at least environmental cost. In these examples, as in the case of building roads to increase food production, our approach cannot provide a definitive guide to infrastructure planning. However, we suggest it could be helpful in assessing where essential, finer-scale follow-up work might be most usefully focused, and in offering a flexible framework for bringing better and additional data together.

## Materials and Methods

### Study Region and Scale

Our study area comprised all of the 2.3 million km^2^ Greater Mekong Subregion (defined by the ADB as Vietnam, Lao PDR, Cambodia, Thailand, Myanmar, and Yunnan Province, China). The GMS is densely settled (with over 320 million inhabitants), culturally diverse, and biologically exceptional (with ~20,000 plant species, 2,000 terrestrial vertebrates, and 850 freshwater fish, many of them endemic) [8,29,42]. Taken together, natural habitats now cover around 59.4% of their original extent [43] and remain vital for generating ecosystem services both globally (e.g., via carbon storage—[44]) and regionally (with the Lower Mekong, for example, supporting the world’s largest inland fishery—[29]). Yet, habitat conversion has been accelerating, with an estimated 31% of natural forest cover in the GMS (excluding Yunnan) lost between 1973 and 2009 [42]. Alongside this, there is widespread poverty, and food insecurity and malnutrition remain major challenges. Faced with continued high population growth and even more rapidly rising food demand, safeguarding remaining natural capital has been recognised as fundamental to achieving Sustainable Development Goals across the GMS [45]. Cooperative and collaborative road networks are a central focus of activities under the GMS banner, but ambitious plans to strengthen the subregion’s transport infrastructure have so far paid scant attention to environmental considerations [8,17].

Analysis was performed at 0.0833° (~10 km x10 km) scale, corresponding to the resolution of the coarsest data layers for the region, and excluded permanent water bodies (as mapped in [46]). In building our aggregate surfaces describing the potential food production benefit and environmental cost of road development, whenever different data layers were added or averaged, we first rescaled them (on a 0–1 scale, with equal-area deciles) to ensure that any outlying values did not have disproportionate influence.

### Potential Food Production Benefit

There are no subregion-wide data on the extent to which poor transport infrastructure limits food production by increasing food waste or production costs. Instead, we took as an aggregate description of how far road improvements might increase local food production through improving yields (and hence without additional habitat conversion) the product of the gap between each cell’s current and attainable production on existing cropland area and its isolation (Fig 1A). To do this, we first generated a surface describing the gap between current and attainable food production on existing cropland (S1A Fig), estimated for each 0.0833° grid cell as the sum (across 17 major crops) of a cell’s yield gap (from [28], converted from tonnes/hectares/y to gigajoules/grid cell/y using [47]; S1 Table) multiplied by its harvested area for that crop [48]. Our analysis excluded some important food and nonfood crops (e.g., *Phaseolus* beans, rubber [49]) and livestock but covers all agricultural products for which yield gaps are given in [28], which together account for 67% of the subregion’s cropland [40]. Including the area of each crop means we estimated the potential for increased production without increasing cropland area and ensures that the yield gap for each crop was weighted by its current areal importance. To incorporate cell isolation, this “production gap” map was then multiplied by travel time (in minutes) to a city of >50,000 people (from [50]; S2 Fig) as an indicator of isolation and hence the scope for road development to improve transport connectivity.

Using this aggregate surface to indicate the potential food production benefit from new roads assumes that each component layer is accurate (an assumption whose effect we explore in our first sensitivity test), that the spatial distribution of production gaps in our sampled crops represents that of all crops and livestock, and that road improvements would have more effect in more isolated areas.

### Potential Environmental Cost

We contrasted this benefit aggregate surface with one reflecting the potential environmental cost of new roads. Our cost surface was a simple mean of three layers describing the impacts which road-induced habitat conversion could be expected to have on biodiversity and the generation of ecosystem services:

1. We estimated importance for terrestrial vertebrates as the proportion of each species’ global range that is within a grid cell, summed across all species in a class (mammals, birds, and amphibians), and multiplied by the estimated proportion of the cell still covered by natural forest, grassland, or shrub [43]; class-specific totals were then rescaled (from 0 to 1 in equal-area deciles), and each cell’s mean value was estimated across all three classes (S3 Fig). Range maps for mammals and amphibians were obtained from IUCN [51], and bird breeding ranges were provided by BirdLife International [52]. Parts of ranges where species have been extirpated or are not native were excluded, but the maps inevitably contain commission and omission errors [53]. Proportional range size (for species estimated as present, based on using Intersects in the rgeos package in R) was taken as cell area divided by the species’ total range size (calculated in ArcMap 10.2). Our estimates of natural cover were refined by removing rubber, oil palm, and timber plantations (using Landsat imagery from the World Agroforestry Centre).
2. We quantified the contribution of each cell’s remaining natural habitats to carbon storage as the carbon loss likely if they were converted to agricultural use, which in the GMS is largely rice production. We estimated carbon loss by combining global maps (in tonnes/hectares) of above- and below-ground biomass carbon stocks (from NASA; [44]) and soil carbon (from [54]; S4 Fig). We assumed that land-use conversion results in the loss of all biomass carbon and a 10% gain in carbon present in the upper 30 cm of soil (as a consequence of postconversion management as rice paddy—after IPCC protocols [55]).
3. As a measure of the importance of natural vegetation for a locally important ecosystem service—biophysical or local climate regulation—we used data from an assessment of how land-cover change affects the loading of heat (via changes to sensible heat flux) and moisture (via changes to latent heat flux) into the atmosphere, adjusting for the relative contribution of wind-transported heat and moisture [56]. This estimated the change in energy balance when natural vegetation is completely removed, expressed as a Heat Regulation Index (in °C) and a Moisture Regulation Index (in mm/d), which reflects downwind precipitation and its impacts on crop production, the timing and flow of streams and rivers, etc. [56,57]. We calculated the mean values of each index for each ecoregion in the study area (from [37,58]), assigned these to each grid cell according to its ecoregion, rescaled the values for each index, multiplied these by the proportion of intact habitat remaining in the cell, and took the mean of these figures. We interpreted the resulting values as indicating how far loss of remaining habitat would alter local-scale climate regulation (S5 Fig).

We then took as our aggregate surface of the potential environmental cost of road expansion the mean of these three layers (each scaled 0–1) (Fig 1B). This step assumes our layers are best combined additively (an assumption whose effect we explore in our second sensitivity test) and that collectively they describe spatial variation in environmental values reasonably well. To examine this latter point, we compared our aggregate surface with the distribution of different priority areas for conservation in the GMS—protected areas, Important Bird Areas, Endemic Bird Areas, Centres of Plant Diversity, Frontier Forests, ADB Conservation Landscapes, Biodiversity Hotspots, Global 200 Ecoregions, and High-Biodiversity Wilderness Areas [30,31,33,36,37].

### Intersecting Potential Benefit and Cost

To shed light on how the relative merits of constructing new roads varied across the GMS, we then used a dual-colour system to intersect our potential food production benefit and environmental cost surfaces (each on a 0–1 scale) (Fig 2). We identified areas where new transport infrastructure would have either relatively high or low potential to increase food production and either marked or more moderate likelihood of harming biodiversity and ecosystem services.

### Comparison with Global Road-Planning Strategy

In broad terms, our framework mirrors the initial global-scale approach [15]. The information contained in some layers has been increased—by including 17 (cf. 4) crops in our yield gap map and all (cf. just threatened) species in our mammal, bird, and amphibian maps. However, in general we used fewer layers, combined them more simply, and dropped those that might be considered redundant (e.g., agro-ecological suitability, already incorporated in the yield gap map [28], and multiple conservation priority maps, themselves largely derived from information incorporated elsewhere on species distributions and habitat extent).

### Existing Road Network

Our approach also improved on the global-scale analysis by considering information on existing infrastructure. This is important because the benefit to food production of improving roads is likely to depend on the current road network (with gains less likely—other things being equal—in areas which already have many roads). We developed a map of the known distribution of existing roads by combining data from Open Street Map [59,60] with government websites (for Thailand and Yunnan only). This was checked with imagery from Google Maps [61], and apparent errors were corrected. We classified roads into four simplified categories (from highways to footpaths), and after excluding Level 4 roads (tracks and footpaths not easily accessible to vehicular traffic) we extracted a surface of road density (expressed as total road length in km / km^2^) based on roads in Levels 1–3 (S6 Fig). This showed that existing roads are clustered around major cities and are sparse in most rural areas.

### Sensitivity Analyses

We undertook three tests of how much our assessment of the costs and benefits of road development depended on the layers we used and our procedures for combining them.

1. Given that individual layers inevitably contain errors, we explored the consequences of using a different layer for the same attribute by using an alternative, independently-derived map of production gaps that integrates yield gaps and area under production for a marginally different set of 16 crops ([40], again converted to gigajoules/hectare/y using data on crop energy content from [47]) (S1B Fig and S1 Table).
2. As an illustration of the effect of combining related layers in different ways, we substituted our potential environmental cost surface based on the mean score across our three component layers with each cell’s maximum value for any of the three layers.
3. Roads are obviously constructed for many reasons unconnected to enhancing food production. So, to examine how far our findings were the result of focusing on food production, we considered more general impacts of road development on human wellbeing by substituting our surface of potential food production benefit with a surface describing human population density (mapped for the year 2000, from [62]; S7 Fig) multiplied by travel time [50]. This gave for each cell a measure of the number of people who might benefit from new roads (for example, by being more easily able to travel for social or economic reasons).

In each case, the substitutions affected only the cost or the benefit aggregate surface, so their effect could be mapped as the difference between each cell’s new and original scores for that surface on a scale of –1 to 1.

### Specific Road Proposals

Finally, we used our framework to assess the arguments for specific developments by mapping proposals for new or improved roads [61] onto our intersection of potential environmental cost and food production benefit (Fig 2) and by calculating mean values of both aggregate surfaces and of travel time, population density, and density of existing roads in a 10 km buffer on either side of each proposed road (S8A Fig and S2 Table). We chose 10 km to match the resolution of our analysis and because this was intermediate between the distance over which meta-analysis indicates roads have population-level effects on mammals (5 km; [12]) and the buffer width used in a recent exercise mapping the overall footprint of major transport corridors (25 km on either side; [27]). To explore differences in how the likely impacts of roads vary along their length, we also plotted out the potential environmental cost and food production benefit values of each 10 km cell adjacent to two individual roads (S8B and S8C Fig).

## Supporting Information

S1 Fig Food production gaps. Based on the Mueller et al. 2012 database [28] (a) and the IIASA/FAO 2012 database [40] (b). Gaps between current and attainable food production on existing cropland were estimated as the sum, across 17 (a) or 16 (b) major crops, of the product of each 0.0833o-cell’s yield gap (expressed in energy terms) and its harvested area for that crop [48]. Note that in subsequent analyses the values plotted here were re-scaled to a 0–1 scale, with equal-area deciles. Underlying data can be found in S1 Data.
(EPS)

S2 Fig Travel time. Surface shows travel time (in mins) to cities with >50,000 people (from [50]). Note that in subsequent analyses the values plotted here were re-scaled to a 0–1 scale, with equal-area deciles. We multiplied this measure of isolation by our production gap maps (S1 Fig) to generate an aggregate surface of the potential food production benefit of new or improved roads. Underlying data can be found in S1 Data.
(EPS)

S3 Fig Importance for terrestrial vertebrates. Calculated as the mean, across mammals, birds and amphibians, of the product of total inverse range size of all species present in each cell and its proportion of intact natural vegetation. Underlying data can be found in S1 Data.
(EPS)

S4 Fig Importance for storage of carbon. Map shows the estimated sum of above- and below-ground live carbon, and soil carbon, and assumes conversion of intact natural vegetation to agriculture results in the loss of all live carbon and a 10% gain in the carbon present in the upper 30cm of soil. Underlying data can be found in S1 Data.
(EPS)

S5 Fig Importance of natural vegetation for local climate regulation. Estimate based on an assessment of how land-cover change affects the loading of heat and moisture into the atmosphere [56]. Underlying data can be found in S1 Data.
(EPS)

S6 Fig Existing road network. Density of existing Level 1, 2 and 3 roads, based on [59] and [60]. This helped our interpretation of variation in the potential costs and benefits of new roads (Fig 2) by identifying areas which were already relatively well-served by transport infrastructure. Underlying data can be found in S1 Data.
(EPS)

S7 Fig Population density. Data from [62]. Note that in subsequent analyses the values plotted here were re-scaled to a 0–1 scale, with equal-area deciles. Underlying data can be found in S1 Data.
(EPS)

S8 Fig Benefits and costs of proposed roads. Mean values for the potential food production benefit of grid cells in a 10km buffer around each of 43 proposed new roads or road improvements, plotted against their mean potential environmental cost (a); and values for each grid cell adjacent to roads TH1 (b) and CMB2 (c). See Fig 2 and S2 Table for more details on road locations and characteristics. Underlying data can be found in S1 Data.
(TIF)

S1 Table The crops whose yield gaps we mapped, and their energy content. (DOCX)

S2 Table Summary of 43 road proposals in the Greater Mekong Subregion. Road locations are from [61] and mapped in Fig 2. Mean values for potential food production benefit, environmental cost, travel time to the nearest city of >50,000 people [50], population density [62], and density of existing roads [59,60] are for a 10km buffer on either side of each road.
(DOCX)

S1 Data Summary of data sources. (XLSX)

S2 Data Underlying GIS files. (ZIP)

## Abbreviations

ADB: Asian Development Bank
GMS: Greater Mekong Subregion
