# Supplementary figures and images for: Getting Road Expansion on the Right Track: A Framework for Smart Infrastructure Planning in the Mekong
Source: PLoS Biol. 2016 Dec 15;14(12):e2000266. doi: 10.1371/journal.pbio.2000266 (PMC5169357; doi:10.1371/journal.pbio.2000266)

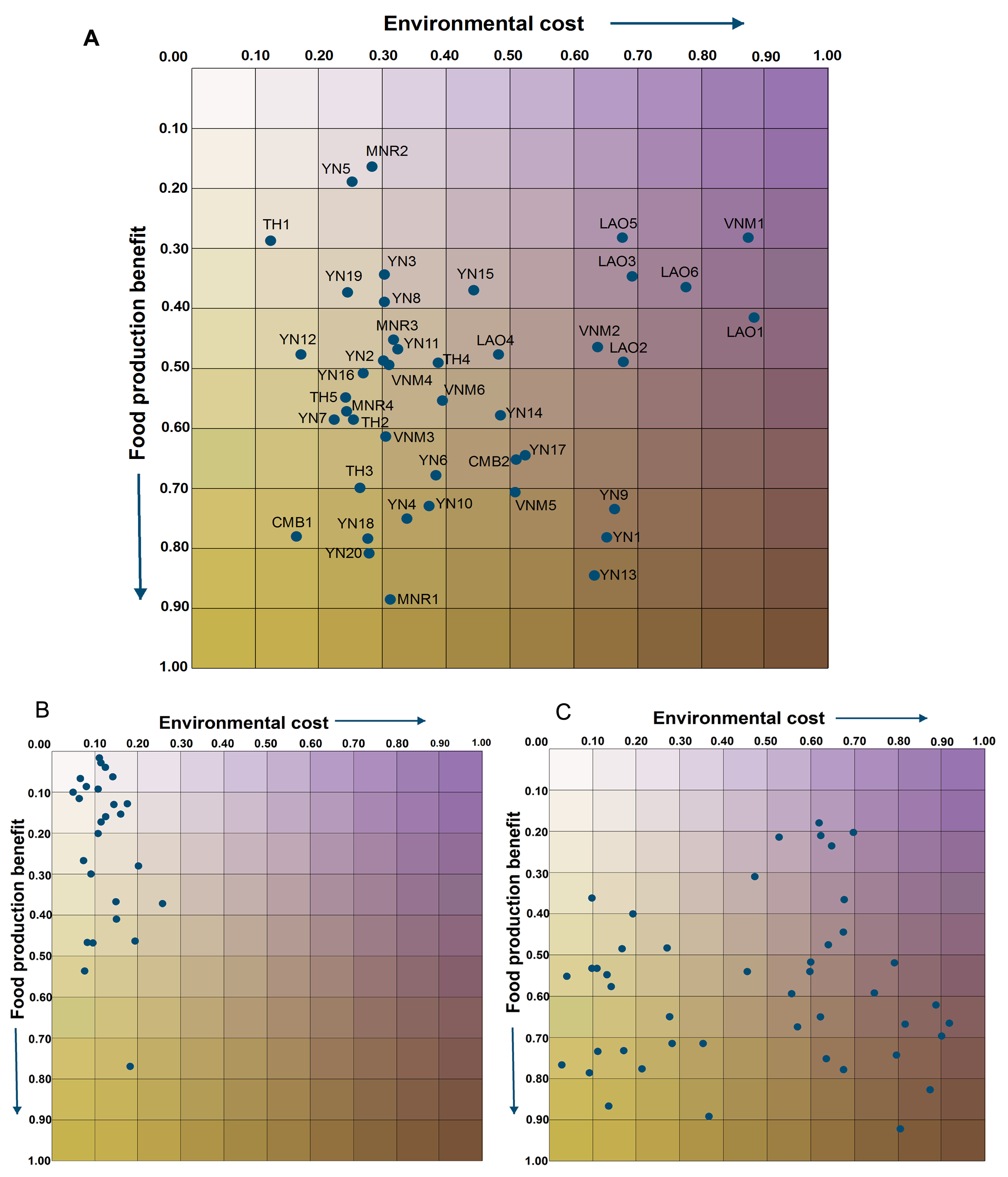

Supplement: S8 Fig — Mean values for the potential food production benefit of grid cells in a 10km buffer around each of 43 proposed new roads or road improvements, plotted against their mean potential environmental cost (a); and values for each grid cell adjacent to roads TH1 (b) and CMB2 (c). See Fig 2 and S2 Table for more details on road locations and characteristics. Underlying data can be found in S1 Data. (TIF) [file pbio.2000266.s008.tif]

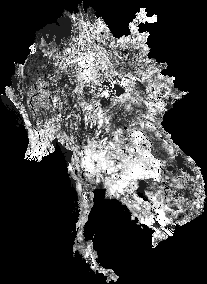

Supplement: S2 Data — (ZIP) [file pbio.2000266.s012.zip › data for uploading_18Oct/S2 Data/intersection of potential food production benefit and environmental cost.tif]

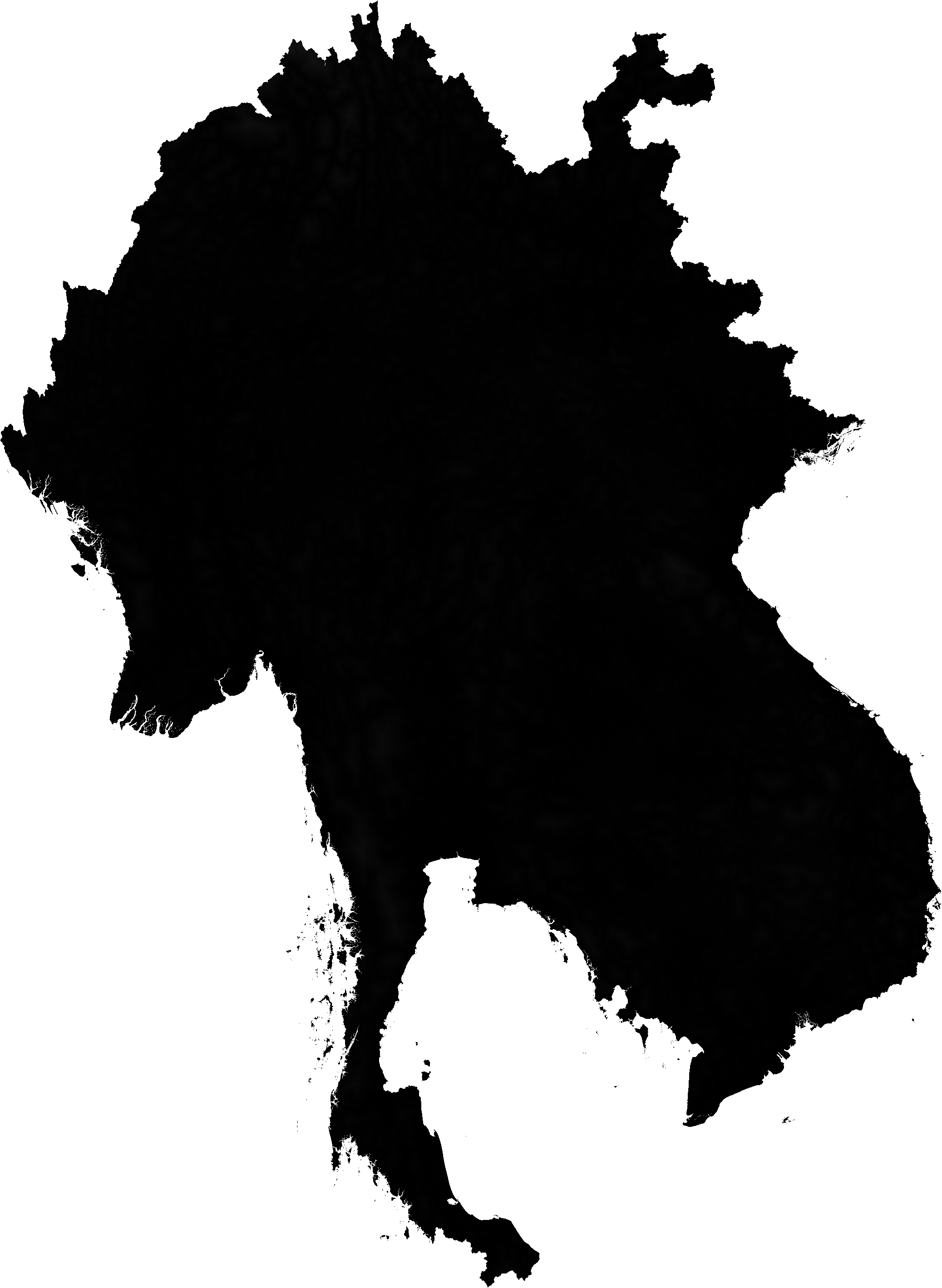

Supplement: S2 Data — (ZIP) [file pbio.2000266.s012.zip › data for uploading_18Oct/SS2 Data/travel_time.tif]
