# Supplementary material for: Getting Road Expansion on the Right Track: A Framework for Smart Infrastructure Planning in the Mekong
Source: PLoS Biol. 2016 Dec 15;14(12):e2000266. doi: 10.1371/journal.pbio.2000266 (PMC5169357; doi:10.1371/journal.pbio.2000266)
Supplement: S1 Table — (DOCX) [file pbio.2000266.s009.docx]

| Crop | Mueller *et al.* (2012) [24] | IIASA/  FAO (2012) [36] | Energy content (GJ / tonne) [44] |
| --- | --- | --- | --- |
| Barley | √ | ✕ | 14.73 |
| Cassava | √ | √ | 5.76 |
| Ground nut | √ | √ | 23.72 |
| Maize | √ | √ | 15.27 |
| Millet | √ | √ | 15.82 |
| Oil palm | √ | √ | 7.40 |
| Potato | √ | √ | 2.62 |
| Rape seed | √ | √ | 14.80 |
| Rice | √ | √ | 15.20 |
| Rye | √ | ✕ | 14.14 |
| Sorghum | √ | √ | 13.77 |
| Soybean | √ | √ | 18.66 |
| Sugar beet | √ | √ | 2.75 |
| Sugarcane | √ | √ | 1.62 |
| Sunflower | √ | √ | 24.44 |
| Wheat | √ | √ | 14.00 |
| Other cereal* | ✕ | √ | 14.71 |

*We estimated the energy content of Other cereal as the mean of that of Barley, Oats, Rye, Triticale and Buckwheat.
