# Supplementary material for: Getting Road Expansion on the Right Track: A Framework for Smart Infrastructure Planning in the Mekong
Source: PLoS Biol. 2016 Dec 15;14(12):e2000266. doi: 10.1371/journal.pbio.2000266 (PMC5169357; doi:10.1371/journal.pbio.2000266)
Supplement: S2 Table — Road locations are from [61] and mapped in Fig 2. Mean values for potential food production benefit, environmental cost, travel time to the nearest city of >50,000 people [50], population density [62], and density of existing roads [59,60] are for a 10km buffer on either side of each road. (DOCX) [file pbio.2000266.s010.docx]

|  | | | | | | | | |
| --- | --- | --- | --- | --- | --- | --- | --- | --- |
| Code | Route | Length (km) | New/  upgrade | Potential food production benefit  (0-1) | Potential environmental cost (0-1) | Travel time (mins) | Population density (people / km^2^) | Density of existing roads (km/ km^2^) |
| MNR1 | Maubin to Phyrapon | 47 | upgrade | 0.89 | 0.31 | 165 | 344 | 0.592 |
| MNR2 | Eindu to Kawkareik | 70 | upgrade | 0.17 | 0.27 | 214 | 120 | 0.160 |
| MNR3 | Tamu to Kalewa to Kalemyo | 159 | upgrade | 0.49 | 0.31 | 421 | 71 | 0.174 |
| MNR4 | Yangon to Mandalay | 589 | upgrade | 0.59 | 0.26 | 152 | 393 | 0.174 |
| TH1 | Bang Yai to Kanchanaburi | 107 | new | 0.29 | 0.12 | 37 | 488 | 0.200 |
| TH2 | Chiang Rai to Chiang Khong | 113 | upgrade | 0.62 | 0.31 | 145 | 104 | 0.972 |
| TH3 | Saraburi to Lomsak | 55 | upgrade | 0.70 | 0.26 | 249 | 109 | 0.170 |
| TH4 | Tak to Mae Sariang | 82 | upgrade | 0.50 | 0.38 | 344 | 67 | 0.216 |
| TH5 | Bang Pa to Nakhon Ratchasima | 196 | new | 0.55 | 0.24 | 74 | 222 | 0.204 |
| CMB1 | Phnom Penh to Poipet | 407 | upgrade | 0.78 | 0.16 | 134 | 330 | 0.520 |
| CMB2 | Phnom Penh to Sihanoukville | 209 | upgrade | 0.66 | 0.50 | 157 | 384 | 0.416 |
| LAO1 | Pakkading to Keoneua | 132 | upgrade | 0.42 | 0.89 | 410 | 11 | 0.123 |
| LAO2 | Ngeun to Pang Hok | 147 | upgrade | 0.49 | 0.68 | 323 | 18 | 0.133 |
| LAO3 | Vientiane to Nateuy | 660 | upgrade | 0.36 | 0.69 | 214 | 78 | 0.267 |
| LAO4 | Vientiane to Veunkham | 853 | upgrade | 0.48 | 0.48 | 204 | 65 | 0.211 |
| LAO5 | Pathoumphon to Phoukeua | 218 | upgrade | 0.28 | 0.68 | 346 | 12 | 0.144 |
| LAO6 | Huayxai to Boten | 225 | upgrade | 0.37 | 0.78 | 288 | 19 | 0.093 |
| VNM1 | Thanh My to Quang Nam | 114 | upgrade | 0.28 | 0.87 | 243 | 21 | 0.114 |
| VNM2 | Sa Pa to Cam Duong | 36 | new | 0.47 | 0.64 | 302 | 195 | 0.367 |
| VNM3 | Ben Luc to Long Thanh | 68 | new | 0.58 | 0.24 | 45 | 3008 | 3.723 |
| VNM4 | Ha Noi to Huu Nghi | 185 | new | 0.46 | 0.32 | 96 | 1094 | 0.797 |
| VNM5 | Do Len to Na Meo | 181 | upgrade | 0.71 | 0.50 | 226 | 288 | 0.144 |
| VNM6 | Bangkok to Namcan | 1042 | upgrade | 0.55 | 0.39 | 184 | 447 | 0.218 |
| YN1 | Baoshan to Lushui | 88 | new | 0.78 | 0.65 | 346 | 111 | 0.185 |
| YN2 | Chenggong to Chengjiang | 34 | new | 0.49 | 0.30 | 108 | 216 | 0.756 |
| YN3 | Funing to Napo | 44 | new | 0.34 | 0.30 | 143 | 120 | 0.373 |
| YN4 | Gongshan to Daibu | 71 | new | 0.76 | 0.34 | 379 | 146 | 0.318 |
| YN5 | Huangtupo to Majinpu | 46 | new | 0.19 | 0.25 | 26 | 1775 | 2.807 |
| YN6 | Huaping to Lijiang | 219 | new | 0.68 | 0.38 | 358 | 109 | 0.349 |
| YN7 | Jiangdi to Zhaokua | 130 | new | 0.59 | 0.22 | 170 | 183 | 0.531 |
| YN8 | Jinning to Hongta | 117 | new | 0.39 | 0.30 | 166 | 658 | 0.832 |
| YN9 | Longling to Ruili | 153 | new | 0.74 | 0.66 | 330 | 134 | 0.462 |
| YN10 | Mazhao Highway | 105 | new | 0.74 | 0.37 | 223 | 293 | 0.619 |
| YN11 | Mengzi to Wenshan | 175 | new | 0.48 | 0.32 | 96 | 202 | 0.387 |
| YN12 | Pingyuan to Wenshan | 41 | new | 0.48 | 0.17 | 83 | 59 | 0.508 |
| YN13 | Ruili to Longchuan | 31 | new | 0.85 | 0.63 | 407 | 144 | 0.193 |
| YN14 | Shangrila to Lijiang | 279 | new | 0.58 | 0.48 | 482 | 66 | 0.404 |
| YN15 | Shiping to Yuanjiang | 122 | new | 0.37 | 0.45 | 131 | 121 | 0.498 |
| YN16 | Songming to Kunming | 42 | new | 0.51 | 0.27 | 83 | 448 | 0.259 |
| YN17 | Xinlin Highway | 521 | new | 0.65 | 0.52 | 340 | 66 | 0.544 |
| YN18 | Xuanwei to Puli | 105 | new | 0.79 | 0.28 | 309 | 239 | 0.620 |
| YN19 | Yangjie to Jijie | 31 | new | 0.38 | 0.24 | 66 | 262 | 0.608 |
| YN20 | Zhaotong to Huize | 122 | new | 0.81 | 0.28 | 265 | 141 | 0.206 |
